# Correction: Graph neural fields: A framework for spatiotemporal dynamical models on the human connectome

**DOI:** 10.1371/journal.pcbi.1010224

**Published:** 2022-06-01

**Authors:** Marco Aqil, Selen Atasoy, Morten L. Kringelbach, Rikkert Hindriks

Fig 5 contains an error due to a misalignment of DTI and MRI data. Figs 6, 8 and 9 contain errors due to the unwarranted exclusion of cross-eigenmode covariance from the calculation of the fMRI functional connectivity. Please view the corrected figures here.

**Fig 5:**
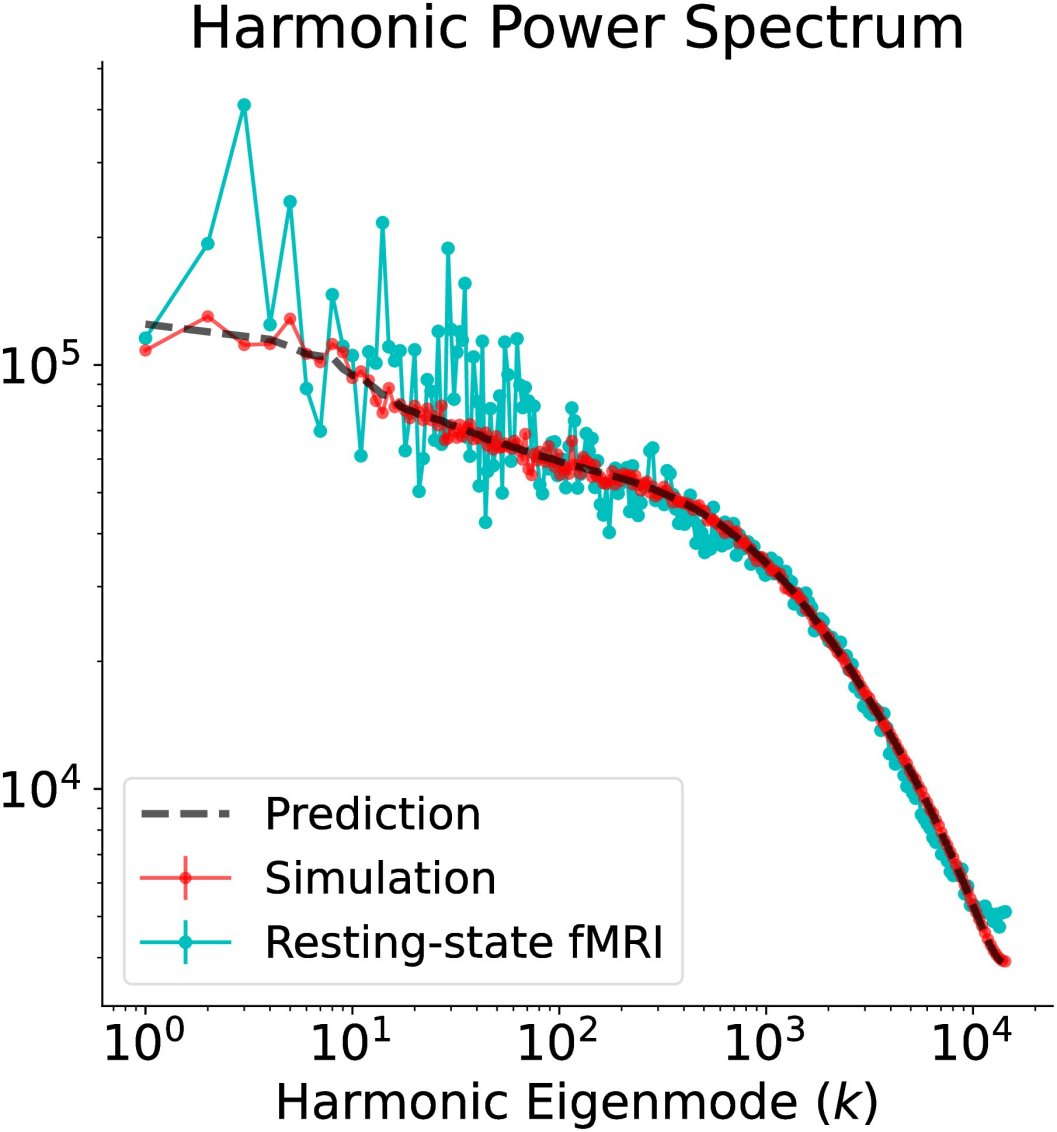
Stochastic Wilson-Cowan graph neural field model captures the resting-state fMRI harmonic power spectrum. The theoretical (dashed black line) and numerical (red line) predictions from the stochastic Wilson-Cowan graph neural field model, with the parameters of S2 Table, are in excellent agreement with the empirically observed fMRI harmonic spectrum (cyan line). The numerical spectrum was obtained by taking the median of three independent simulations.

**Fig 6:**
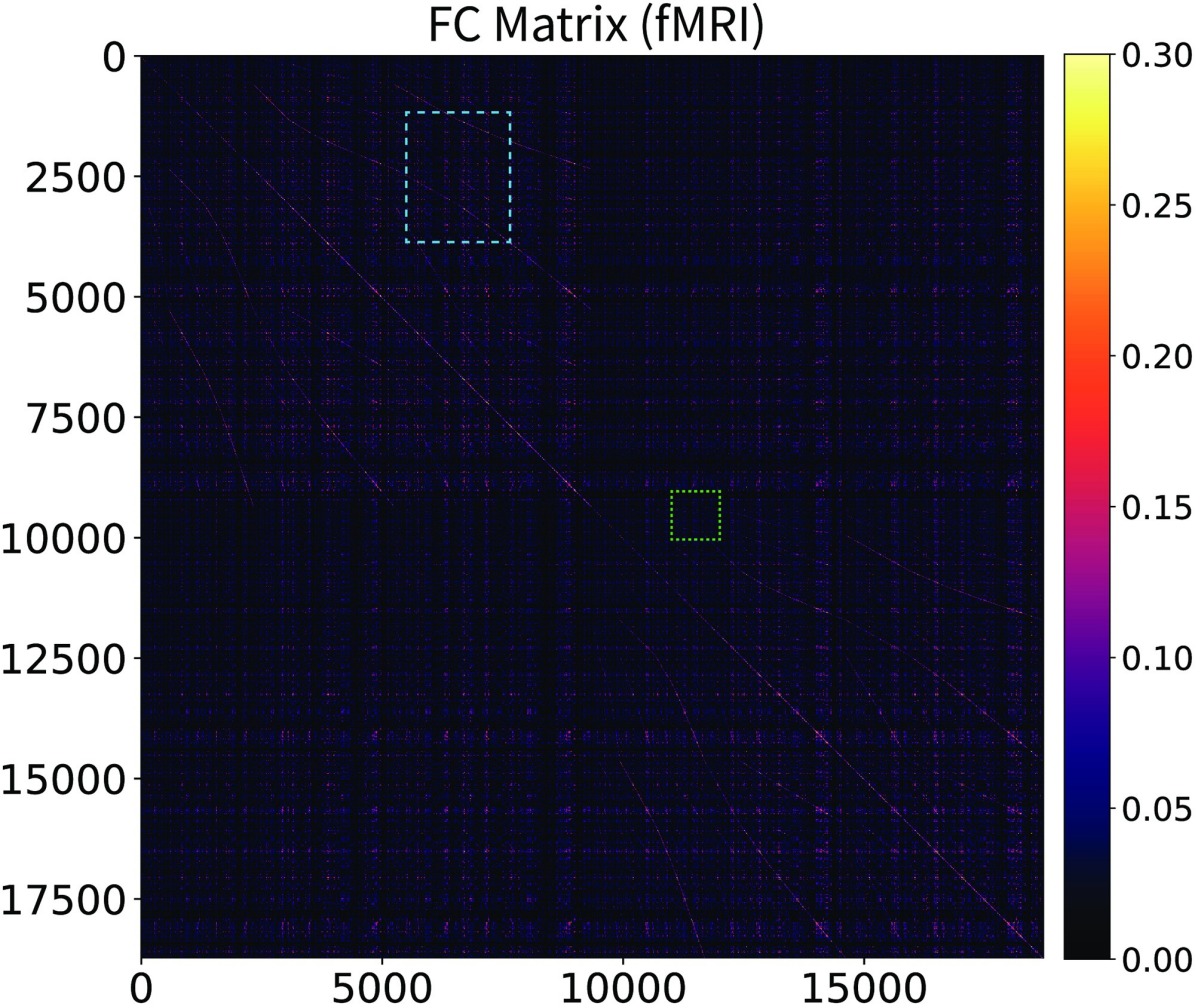
Resting-state fMRI functional connectivity matrix. Connectome-wide, vertex-wise, single-subject, resting-state fMRI functional connectivity matrix. Zoom in to appreciate the patterns present in the data, in particular the two large blocks (top-left and bottom-right) corresponding to the two hemispheres, and the many intra-hemispheric patterns. Compare with the functional connectivity predicted by the stochastic Wilson-Cowan graph neural field (Fig 7). The light-blue and light-green rectangles indicate the insets visualized in Figs 8 and 9.

**Fig 8:**
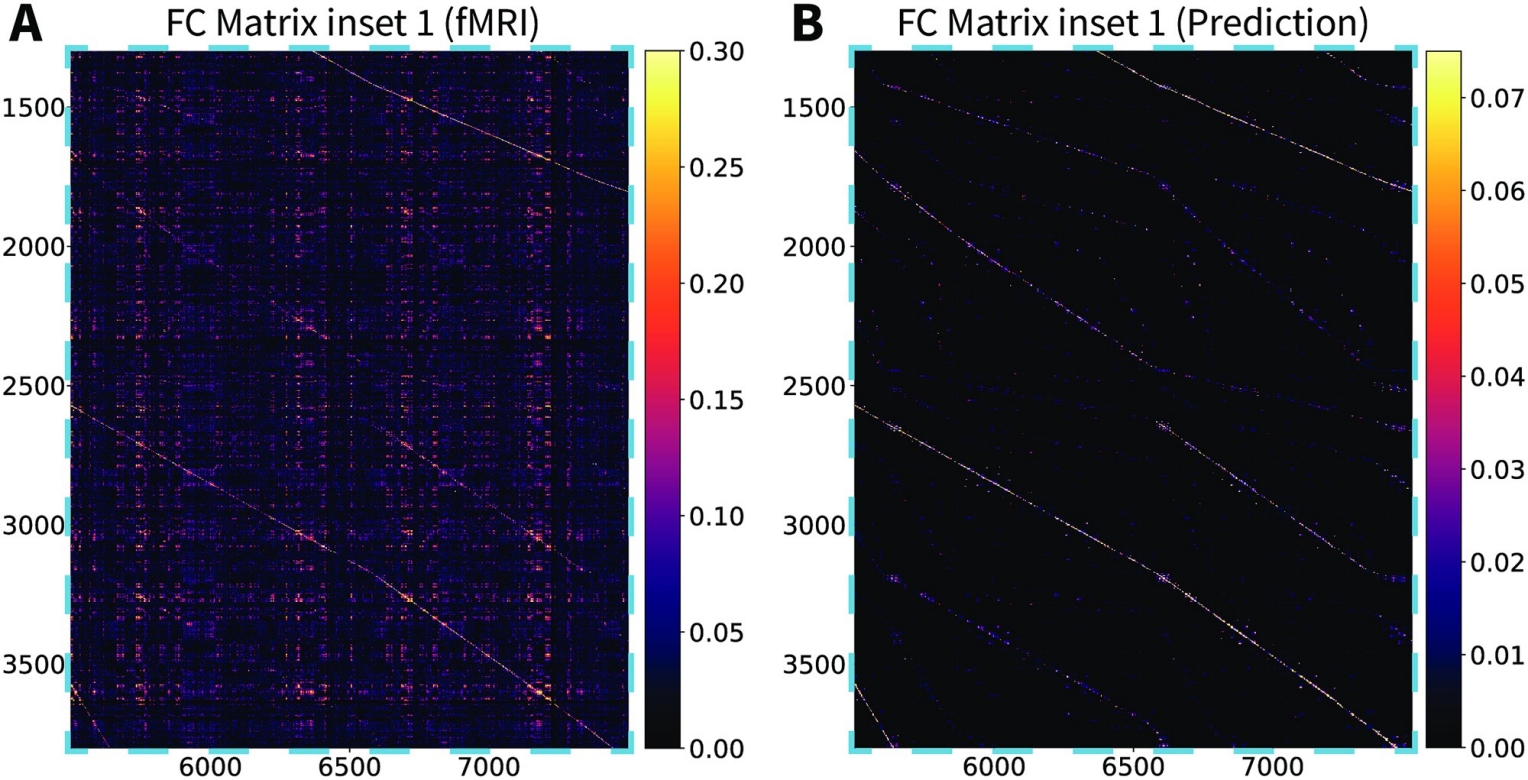
Stochastic Wilson-Cowan graph neural field model predicts the experimental functional connectivity matrix (inset 1). (A) An inset of the vertex-wise, resting-state fMRI functional connectivity matrix for a single subject. (B) The same inset for the Wilson-Cowan graph neural field model with the parameters of S2 Table.

**Fig 9:**
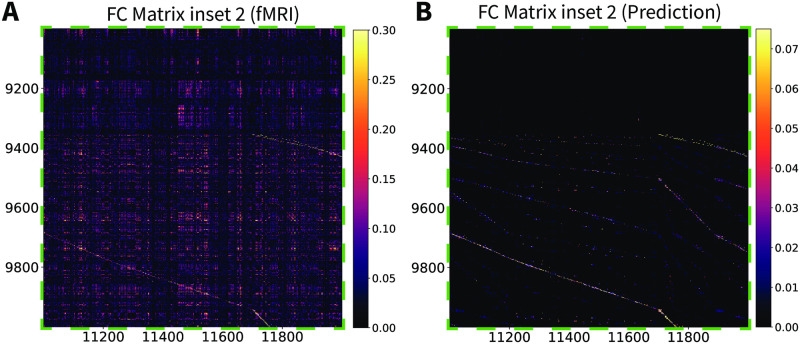
Stochastic Wilson-Cowan graph neural field model predicts the experimental functional connectivity matrix (inset 2). (A) An inset of the vertex-wise, resting-state fMRI functional connectivity matrix for a single subject. (B) The same inset for the Wilson-Cowan graph neural field model with the parameters of S2 Table.

There is a typographical error in S1 Table. The value 7.903 ∙ 10^2^ should read 7.903 ∙ 10^1^. Please view the corrected table here.

## Supporting information

S1 Table Parameter set for 1D analysis and simulations. This parameter set was obtained by a qualitative comparison of the Wilson-Cowan model’s harmonic and temporal spectra with empirical data, and used to illustrate how graph properties affect neural field dynamics in one dimension.
(PDF)

## References

[1] Aqil M, Atasoy S, Kringelbach ML, Hindriks R (2021) Graph neural fields: A framework for spatiotemporal dynamical models on the human connectome. PLOS Computational Biology 17(1): e1008310. doi: 10.1371/journal.pcbi.1008310 33507899 PMC7872285

