# Supplementary material for: Graph neural fields: A framework for spatiotemporal dynamical models on the human connectome
Source: PLoS Comput Biol. 2021 Jan 28;17(1):e1008310. doi: 10.1371/journal.pcbi.1008310 (PMC7872285; doi:10.1371/journal.pcbi.1008310)
Supplement: S2 Table — This parameter set was obtained by quantiatively fitting the Wilson-Cowan model’s harmonic power spectrum to that of resting-state fMRI data, and used for all connectome-wide analysis and numerical simulations. (PDF) [file pcbi.1008310.s002.pdf]

Parameter set for connectome-wide analysis and simulations.

Marco Aqil, Selen Atasoy, Morten L. Kringelbach, Rikkert Hindriks

November 26, 2020

**Parameter**	**Value**	**Units (S.I)**
τ_E	$2.024 \cdot 10^{-1}$	s
τ_I	$2.346 \cdot 10^{-1}$	s
σ_{EE}	$1.611 \cdot 10^{-2}$	m
σ_{IE}	$2.022 \cdot 10^{-3}$	m
σ_{EI}	$6.698 \cdot 10^{-2}$	m
σ_{II}	$9.149 \cdot 10^{-2}$	m
d_E	$2.718 \cdot 10^{1}$	-
d_I	1.240	-
α_{EE}	$1.487 \cdot 10^{2}$	-
α_{IE}	$2.191 \cdot 10^{2}$	-
α_{EI}	$2.620 \cdot 10^{2}$	-
α_{II}	$1.614 \cdot 10^{2}$	-
P	$2.235 \cdot 10^{1}$	-
Q	8.450	-
σ	10^{-7}	-
