# Supplementary figures and images for: Graph neural fields: A framework for spatiotemporal dynamical models on the human connectome
Source: PLoS Comput Biol. 2021 Jan 28;17(1):e1008310. doi: 10.1371/journal.pcbi.1008310 (PMC7872285; doi:10.1371/journal.pcbi.1008310)

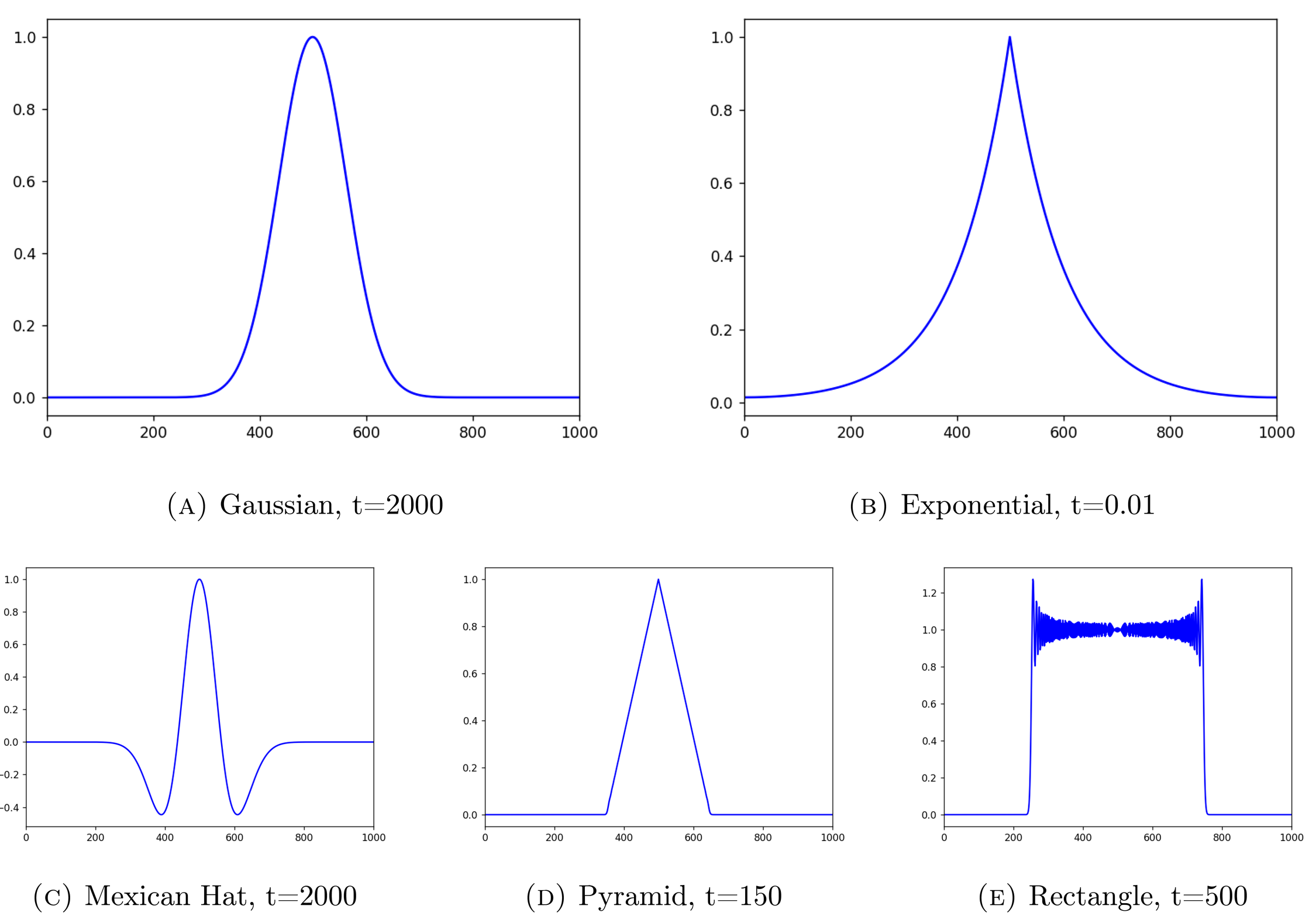

Supplement: S1 Fig — To illustrate spatial convolution on graphs, we apply different spatial convolution filters from Table 2 to an impulse function centered on the middle vertex of a one-dimensional grid-graph with spacing h = 1 units. The resulting functions, normalized to have unit amplitude, show the shapes of the graph kernels. Note that the rectangular kernel convolution operator in Panel (E) exhibits the Gibbs phenomenon [64], which is a known feature of finite Fourier representations of functions with jump discontinuities. Solutions to this problem have been offered [65], but they are beyond the scope of the current work. Thus, we suggest avoiding spatial kernels with jump discontinuities in the context of graph neural fields. Open boundary conditions can be implemented by extending the graph beyond the image size, and periodic boundaries by adding edges connecting vertices on opposite sides of the graph. We also note that, if desired, spectral kernels can be obtained using polynomial approximation schemes, which obviates the need to diagonalize the graph Laplacian matrix [66]. For large datasets (for example natural images databases), it might be computationally advantageous to apply convolutions with symmetric kernels through graph filters, rather than with standard discrete convolution methods. Blurring/smoothing a 2-dimensional image with a spatial Gaussian kernel is equivalent to applying the graph Gaussian kernel to the image-function defined on a 2-dimensional square-grid graph. Spatial convolutions on graphs become linear matrix-vector products, which are highly optimized and easily parallelizable operations; the bulk of the computational cost for graph convolutions consists in the initial computation of the filter itself, which has to be performed only once per kernel. The approach described here is limited to symmetric kernels. In some special cases, asymmetric kernels may be practically obtained by introducing suitable asymmetries in the graph edges. For example, cons [file pcbi.1008310.s003.tif]

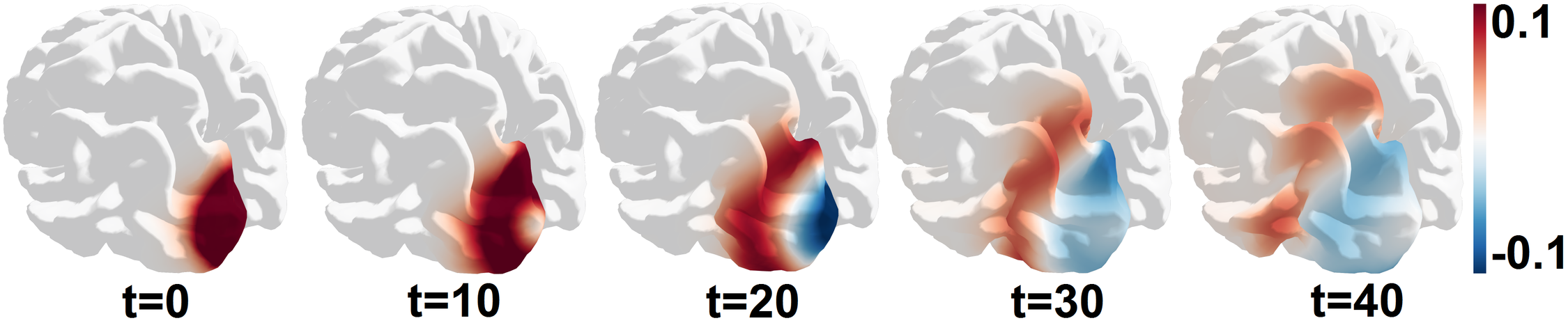

Supplement: S2 Fig — Shown are snapshots of simulated cortical activity that is governed by the damped wave equation with time-step δt = 1 and parameters a = 3 ⋅ 105, b = 5 ⋅ 103. (TIF) [file pcbi.1008310.s004.tif]

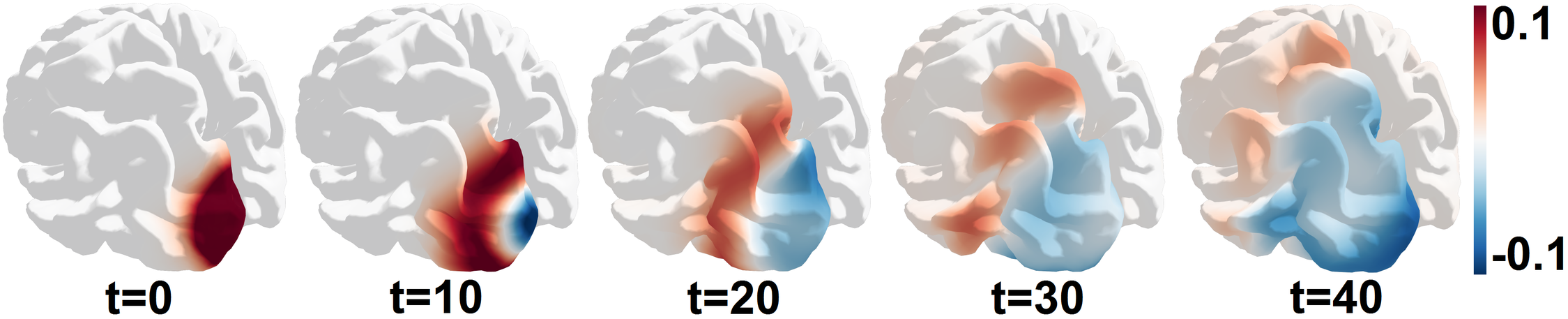

Supplement: S3 Fig — Shown are snapshots of simulated cortical activity that is governed by the damped wave equation with time-step δt = 1 and parameters a = 1.5 ⋅ 105, b = 2.5 ⋅ 103. (TIF) [file pcbi.1008310.s005.tif]

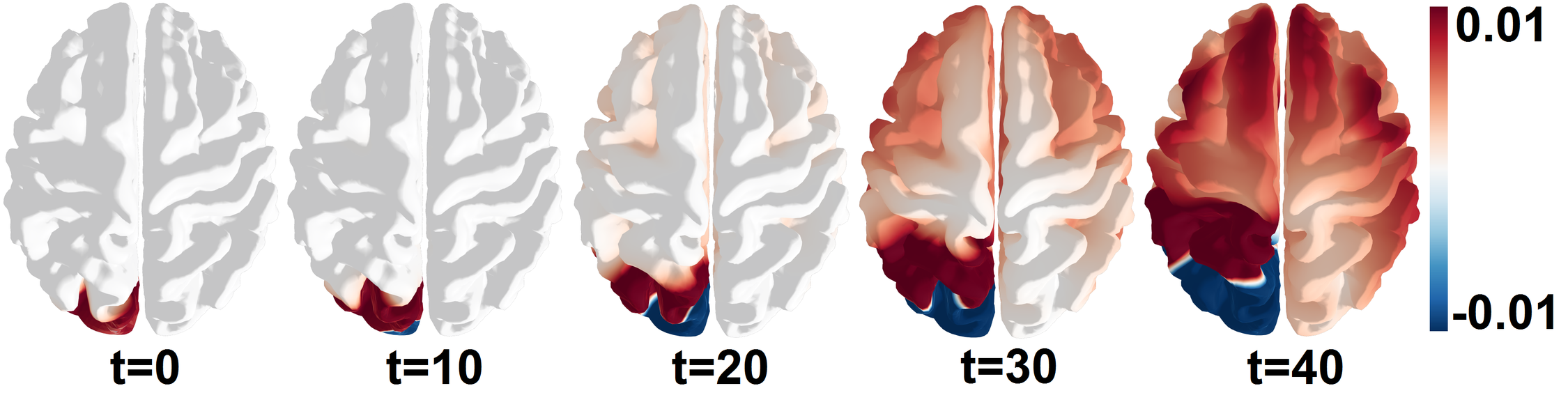

Supplement: S4 Fig — Shown are snapshots of simulated cortical activity that is governed by the damped wave equation with time-step δt = 1 and parameters a = 1.5 ⋅ 105, b = 2.5 ⋅ 103. (TIF) [file pcbi.1008310.s006.tif]

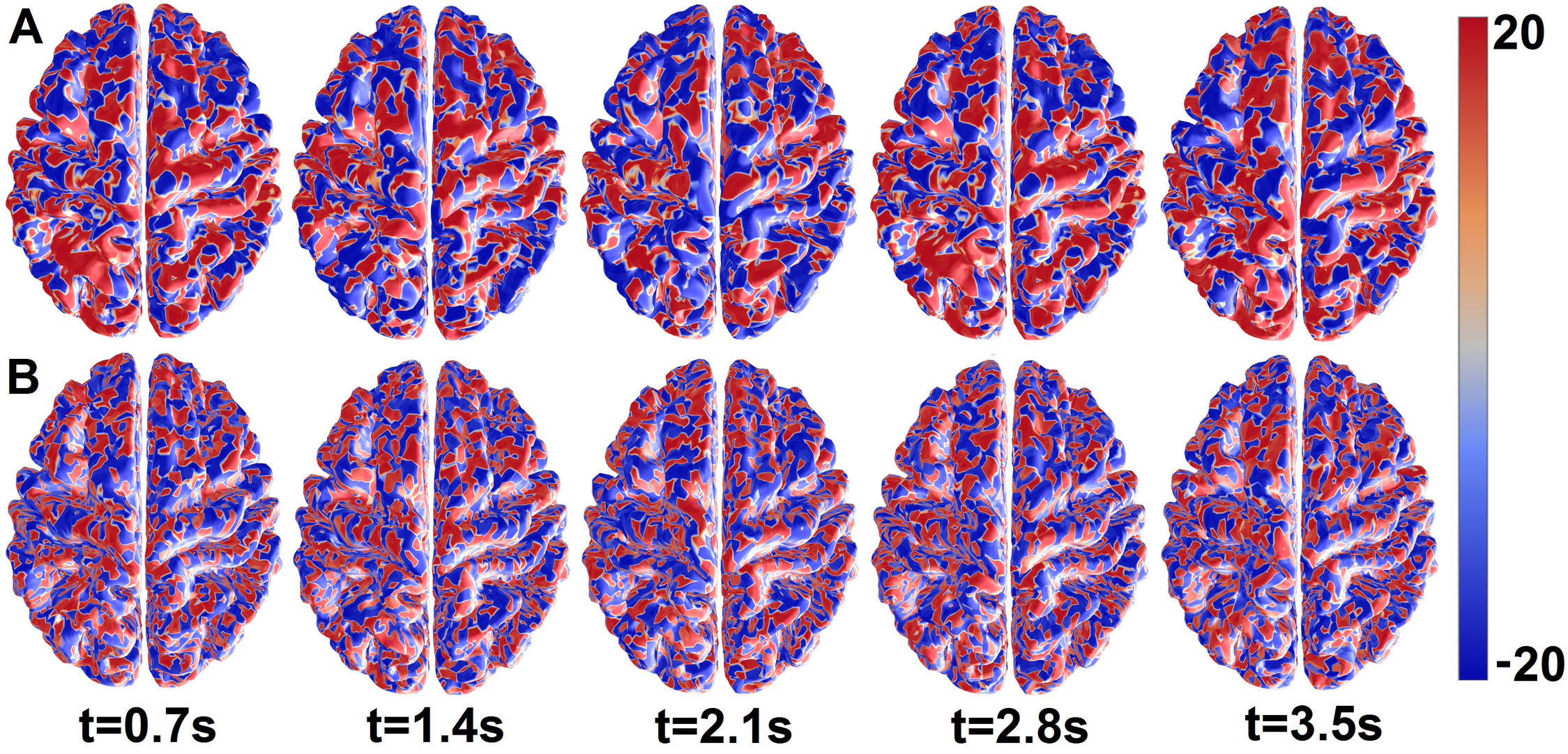

Supplement: S5 Fig — Panel A shows resting-state brain activity, as fluctuations of the BOLD fMRI signal about the mean at each vertex. Panel B shows snapshots of activity from the stochastic Wilson-Cowan graph neural field model, simulated using the parameters of S2 Table. The model activity was temporally downsampled to match the TR of fMRI data, and rescaled by β to match the scale of the BOLD signal. No spatial or temporal smoothing was applied. Note that the two hemispheric surfaces are physically separate, and inter-hemispheric propagation is allowed through white matter fibers. (TIF) [file pcbi.1008310.s007.tif]

FC Matrix (fMRI)

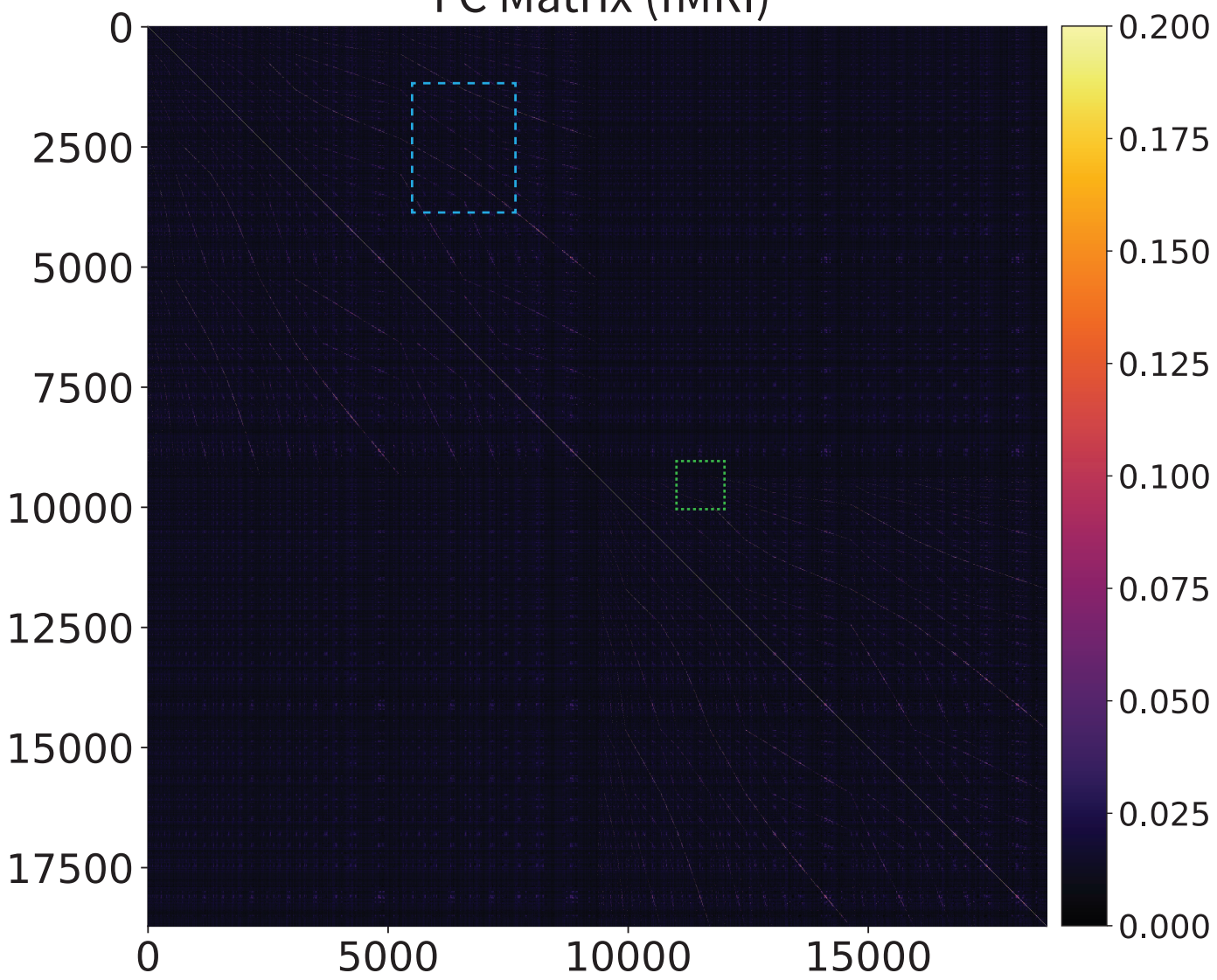

Supplement: S6 Fig — High-resolution PDF version of Fig 6. (PDF) [file pcbi.1008310.s008.pdf]

FC Matrix (Prediction)

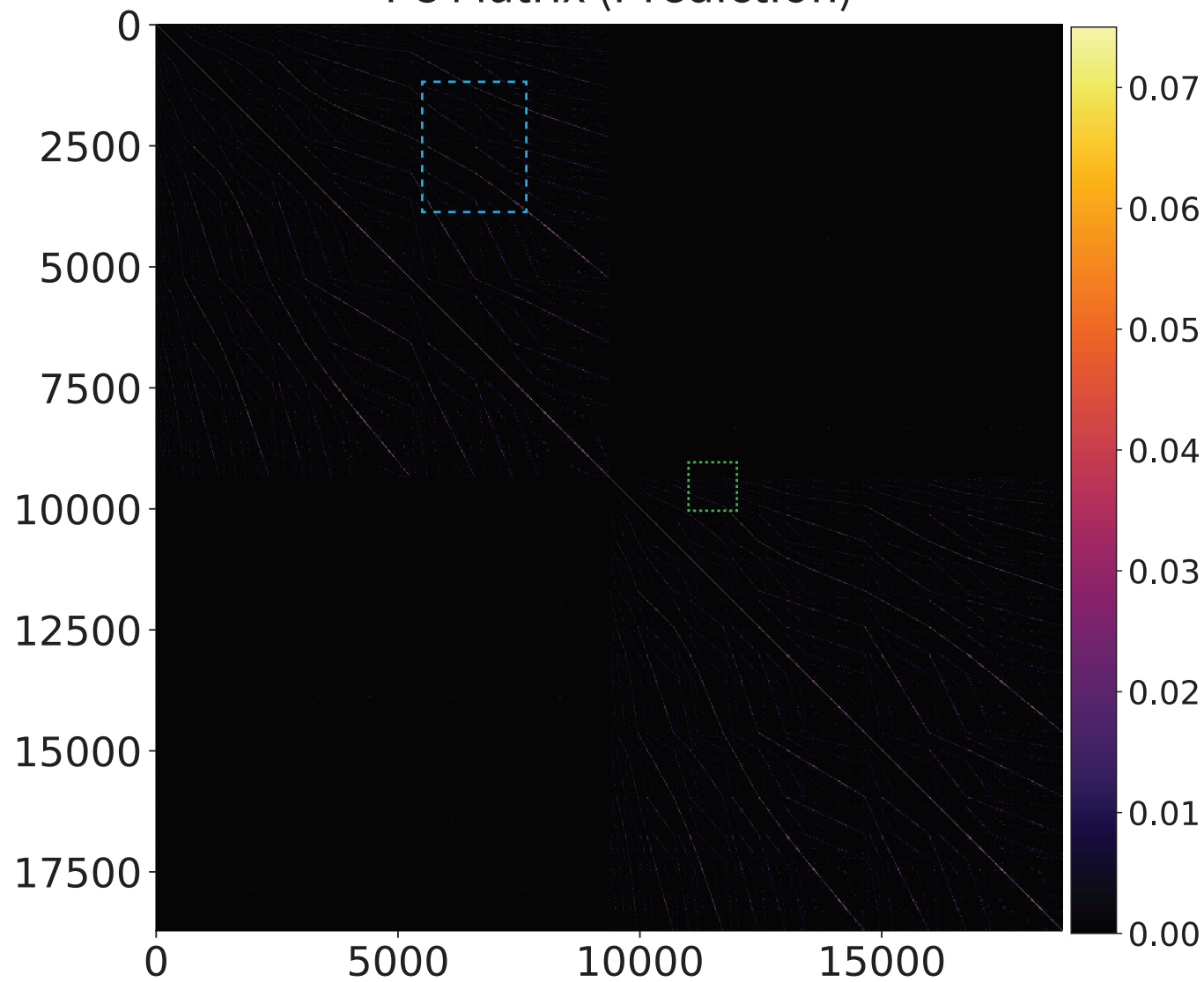

Supplement: S7 Fig — High-resolution PDF version of Fig 7. (PDF) [file pcbi.1008310.s009.pdf]

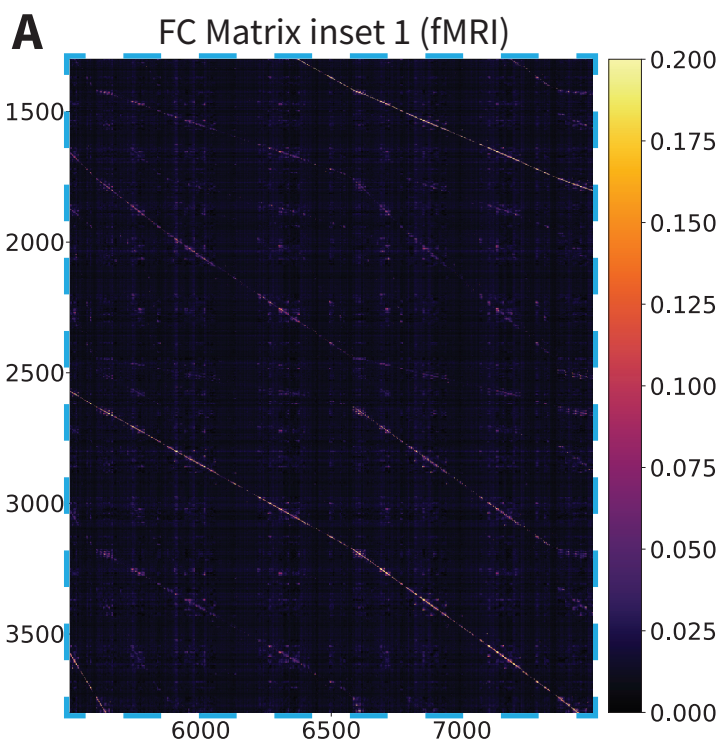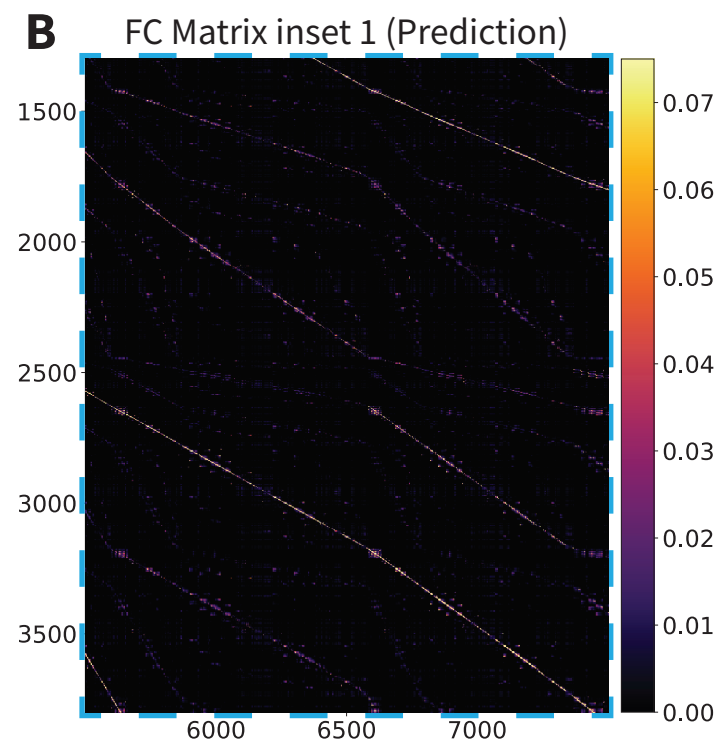

Supplement: S8 Fig — High-resolution PDF version of Fig 8. (PDF) [file pcbi.1008310.s010.pdf]

**A** FC Matrix inset 2 (fMRI)

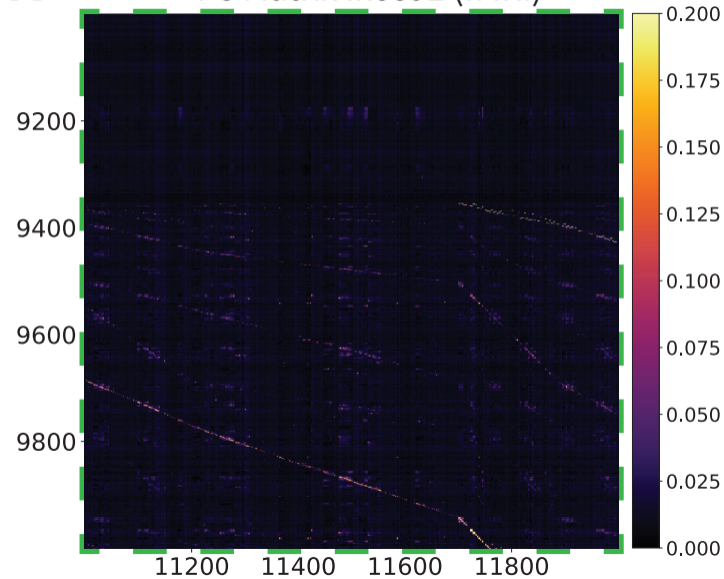

**B** FC Matrix inset 2 (Prediction)

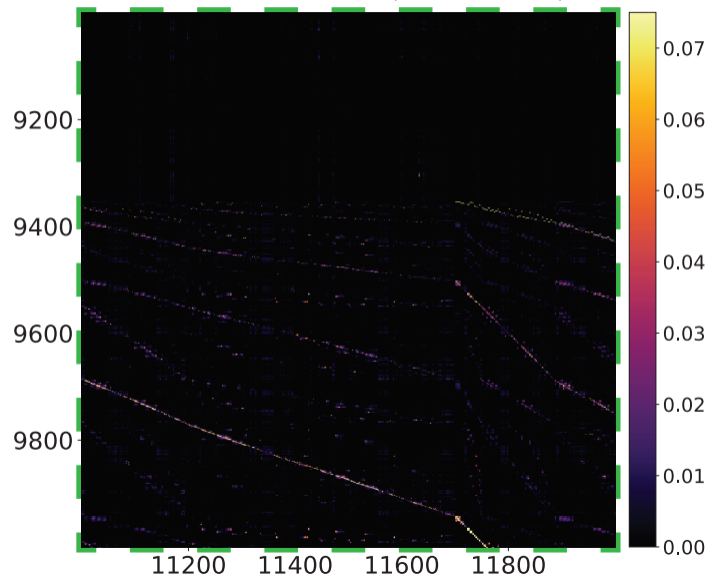

Supplement: S9 Fig — High-resolution PDF version of Fig 9. (PDF) [file pcbi.1008310.s011.pdf]
