# Supplementary material for: Graph neural fields: A framework for spatiotemporal dynamical models on the human connectome
Source: PLoS Comput Biol. 2021 Jan 28;17(1):e1008310. doi: 10.1371/journal.pcbi.1008310 (PMC7872285; doi:10.1371/journal.pcbi.1008310)
Supplement: S1 Appendix — Here, we generalize the formulation of spatial convolutions on graphs to spatiotemporal convolutions on graphs, allowing the definition of a broader class of graph neural fields. (PDF) [file pcbi.1008310.s012.pdf]

Spatiotemporal convolutions on graphs.

Marco Aqil, Selen Atasoy, Morten L. Kringelbach, Rikkert Hindriks

November 26, 2020

Consider the continuous spatiotemporal convolution:

$$(K \otimes u)(x,t) = \int_{-\infty}^{\infty} \int_{-\infty}^{\infty} K(x-x', t-t') u(x', t') dx' dt'. \tag{1}$$

Let $K(x,t)$ be a symmetric, continuous kernel with Fourier transform $\hat{K}(-k^2, \omega)$ and let $u(t)$ be a function on the graph with graph Fourier transform $\hat{u}(t)$. We denote the temporal Fourier transforms of $u(t)$ and $\hat{u}(t)$ by $u(\omega)$ and $\hat{u}(\omega)$, respectively. We define the graph kernel $\hat{K}_g$ associated with the continuous kernel K:

$$\hat{K}_g = \mathrm{Diag}(\hat{K}(\lambda_1, \omega), \cdots, \hat{K}(\lambda_n, \omega)). \tag{2}$$

In the graph Fourier and temporal frequency domains, the filtered signal is hence per definition given by:

$$\hat{u}^{\text{filt}}(\omega) = \hat{K}_g \hat{u}(\omega). \tag{3}$$

Applying the inverse graph Fourier transform U, we obtain the filtered signal in the graph domain:

$$u^{\text{filt}}(\omega) = U\hat{K}_g \hat{u}(\omega) = U\hat{K}_g U^T u(\omega) = K_g u(\omega), \tag{4}$$

where we have defined $K_g = U\hat{K}_g U^T$, the graph domain representation of the filter.

The above representations of the kernel and the filtered signal are in the temporal frequency domain. To obtain the corresponding time domain representations, we note that the ith entry of $u^{\text{filt}}(\omega)$ is given by:

$$u_i^{\text{filt}}(\omega) = \sum_{j=1}^{n} K_g^{i,j}(\omega) u_j(\omega), \tag{5}$$

where $K_g^{i,j}(\omega)$ denotes the (i,j) entry of $K_g(\omega)$. Using the convolution theorem, the time domain representation of the filtered signal is:

$$u_i^{\text{filt}}(t) = \sum_{j=1}^{n} (K_g(i,j) \otimes u_j)(t). \tag{6}$$

Collecting the terms for all n entries in a column vector yields the filtered signal in the graph and temporal domain and we write it as $(K_g \otimes_g u)(t)$:

$$u^{\text{filt}}(t) = (K_g \otimes_g u)(t). \tag{7}$$

In case of a purely spatial kernel $K(x,t) = K(x)$, with $K(x)$ symmetric and with spatial Fourier transform $\hat{K}(-k^2)$, the graph kernel $\hat{K}_g$ is independent of frequency so that the filtering operator reduces to the

frequency-independent linear transformation:

$$(K_g \otimes_g u)(t) = K_g u(t). \tag{8}$$

In case of a purely temporal kernel $K(x,t) = g_\Theta(t)$, with $g_\Theta(t) = g(t)\Theta(t)$, where $g(t)$ is the temporal kernel, and $\Theta(t)$ the Heaviside step function, which ensures that integration is temporally causal. Graph filtering reduces to a temporal convolution:

$$(K_g \otimes_g u)(t) = (g_\Theta \otimes u)(t). \tag{9}$$

Lastly, in case of a separable kernel $K(x,t) = w(x)g_\Theta(t)$, the graph kernel decomposes as:

$$\hat{K}_g = g_\Theta(\omega)\mathrm{Diag}(\hat{w}(\lambda_1), \cdots, \hat{w}(\lambda_n)), \tag{10}$$

so that the filtered signal is given by:

$$(K_g \otimes_g u)(t) = (g_\Theta \otimes (K_g u))(t). \tag{11}$$

In this case, the filtering occurs in two stages: the signal of first spatially filtered by K_g and subsequently temporally filtered by the convolution with $g_\Theta(t)$.
