# Supplementary material for: Graph neural fields: A framework for spatiotemporal dynamical models on the human connectome
Source: PLoS Comput Biol. 2021 Jan 28;17(1):e1008310. doi: 10.1371/journal.pcbi.1008310 (PMC7872285; doi:10.1371/journal.pcbi.1008310)
Supplement: S2 Appendix — In this section we show how graph filters can also be used to implement the graph equivalents of neural activity models that can be directly written as partial differential equations [36, 53] and, among others, comprise damped wave and reaction-diffusion equations. (PDF) [file pcbi.1008310.s013.pdf]

Reaction-diffusion neural activity models on graphs.

Marco Aqil, Selen Atasoy, Morten L. Kringelbach, Rikkert Hindriks

November 26, 2020

Consider the following general reaction-diffusion model:

$$D_t u(x,t) = \frac{\partial^2}{\partial x^2} u(x,t) + \sigma \xi(x,t), \tag{1}$$

where D_t denotes the temporal differential operator that describes local reactions, $\partial^2/\partial x^2$ is the diffusion term and $\sigma\xi(x,t)$ is an external forcing function. To obtain the corresponding graph equation we first transform Eq (1) to the spatiotemporal Fourier domain:

$$D(\omega)\hat{u}(k,\omega) = -k^2 \hat{u}(k,\omega) + \sigma\hat{\xi}(k,\omega), \tag{2}$$

where $D(\omega)$ denotes the Fourier transform of D_t, and subsequently solve for $\hat{u}(k,\omega)$:

$$\hat{u}(k,\omega) = \sigma \hat{K}(k,\omega)\hat{\xi}(k,\omega), \tag{3}$$

where the spatiotemporal kernel $\hat{K}(k,\omega)$ is given by:

$$\hat{K}(k,\omega) = \frac{1}{D(\omega) + k^2}. \tag{4}$$

The corresponding graph kernel is given by:

$$\hat{K}_g = (D(\omega) - \Lambda)^{-1}, \tag{5}$$

where Λ denotes the diagonal matrix containing the eigenvalues of the weighted graph Laplacian Δ. Applying $\hat{K}_g$ to the input gives

$$\hat{u}(\omega) = \sigma \hat{K}_g \hat{\xi}(\omega). \tag{6}$$

Transforming back to the graph-temporal domain to obtain $u(t) = \sigma(K_g \otimes \xi)(t)$. To obtain the full system of differential equations, we note that

$$(D(\omega) - \Lambda)\hat{u}(\omega) = \sigma\hat{\xi}(\omega), \tag{7}$$

and transform this equation back to the spatial domain to obtain the following system of ordinary differential equations:

$$D_t u(t) = \Delta u(t) + \sigma\xi(t). \tag{8}$$

This shows that a reaction diffusion equation can directly be defined on a graph by replacing the Laplace operator in continuous space by the weighted graph Laplacian Δ, and solved by computing a suitable graph

filter. For example, setting $D_t = d/dt$ will give the graph equivalent of the diffusion equation, which is solved by a Gaussian filter; Setting $D_t = ad^2/dt^2$ will give the wave equation; setting $D_t = ad^2/dt^2 + bd/dt$ will give the damped-wave equation, for which we present a solution and implementation on the human connectome graph in *S3 Appendix*.
