# Supplementary material for: Graph neural fields: A framework for spatiotemporal dynamical models on the human connectome
Source: PLoS Comput Biol. 2021 Jan 28;17(1):e1008310. doi: 10.1371/journal.pcbi.1008310 (PMC7872285; doi:10.1371/journal.pcbi.1008310)
Supplement: S3 Appendix — The damped-wave describes the dynamics of simultaneous diffusion and wave propagation, and is thus of interest in the context of modelling activity propagation in neural tissue [53]. Nonlinear variants of the wave equation on graphs have also been the subject of previous analytical studies [67]. Here, we solve the graph equivalent of the damped-wave equation and of the telegrapher’s equation, which is of interest in the context of modelling action potentials [68]. (PDF) [file pcbi.1008310.s014.pdf]

# Damped wave and telegrapher's equation on graphs.

Marco Aqil, Selen Atasoy, Morten L. Kringelbach, Rikkert Hindriks

November 26, 2020

In one-dimensional continuous space, the damped-wave equation is:

$$a\frac{\partial^2 u(x,t)}{\partial t^2} + b\frac{\partial u(x,t)}{\partial t} = \frac{\partial^2 u(x,t)}{\partial x^2}, \tag{1}$$

where $a$ and $b$ are scalar parameters. Its graph-equivalent is:

$$D_t^{DW} u(t) = \Delta u(t), \tag{2}$$

where $u(t)$ is a function on the graph, and the temporal operator for the damped-wave equation is given by $D_t^{DW} = ad^2/dt^2 + bd/dt$. Since the graph Laplacian $\Delta$ is a constant matrix, we can straightforwardly obtain the exact solution at time $t$ in the graph Fourier domain:

$$\hat{u}(t) = K(t)\hat{u}(0) + \tilde{K}(t)\frac{d\hat{u}}{dt}(0), \tag{3}$$

where $(\hat{u}(0), \frac{d\hat{u}}{dt}(0))$ are the initial conditions, and:

$$K(t) = \frac{r_1 e^{r_2 t} - r_2 e^{r_1 t}}{r_1 - r_2}, \qquad \tilde{K}(t) = \frac{e^{r_1 t} - e^{r_2 t}}{r_1 - r_2}, \qquad r_{1,2} = \frac{-b \pm \sqrt{b^2 + 4a\Lambda}}{2a}, \tag{4}$$

Having obtained an exact solution, we can efficiently simulate the time-evolution of the damped-wave equation on arbitrary metric graphs, for example with one of the following numerical schemes (forward and backward approximations of the time derivative of the initial condition):

$$\hat{u}(t+\delta t) = \left(1 - \frac{\tilde{K}(\delta t)}{\delta t}\right)^{-1}\left(K(\delta t) - \frac{\tilde{K}(\delta t)}{\delta t}\right)\hat{u}(t), \tag{5}$$

$$\hat{u}(t+\delta t) = K(\delta t)\hat{u}(t) + \tilde{K}(\delta t)\frac{\hat{u}(t) - \hat{u}(t-\delta t)}{\delta t}. \tag{6}$$

We also note that the telegrapher's equation, which is of interest in the context of modelling action potentials:

$$a\frac{\partial^2 u(x,t)}{\partial t^2} + b\frac{\partial u(x,t)}{\partial t} + cu(x,t) = \frac{\partial^2 u(x,t)}{\partial x^2}, \tag{7}$$

can also be implemented on metric graphs simply by substituting $\Lambda$ with $(\Lambda - \mathrm{Diag}(c))$ in Eq (4).
