# Supplementary material for: Graph neural fields: A framework for spatiotemporal dynamical models on the human connectome
Source: PLoS Comput Biol. 2021 Jan 28;17(1):e1008310. doi: 10.1371/journal.pcbi.1008310 (PMC7872285; doi:10.1371/journal.pcbi.1008310)
Supplement: S4 Appendix — In order to compute meaningful spatiotemporal observables with CHAOSS for a given set of parameters, it is first necessary to find a steady state and compute its stability to perturbations. Here, we provide solutions to the steady-state equations and a general linear stability analysis for the Wilson-Cowan model on graphs. (PDF) [file pcbi.1008310.s015.pdf]

Wilson-Cowan model linear stability analysis.

Marco Aqil, Selen Atasoy, Morten L. Kringelbach, Rikkert Hindriks

November 26, 2020

For generality and compactness of notation, let us define a new column vector u as the concatenation of E and I; we express γ for the diagonal matrix containing the damping parameters d_E and d_I,τ for the diagonal matrix containing the timescale parameters τ_E and τ_I. The matrix K contains the four (arbitrary) graph-filters, X is the concatenation of subcortical input vectors P and Q. We can now write the Wilson-Cowan model on graphs (see main text) with a single equation, in the absence of noise:

$$\frac{du}{dt} = -\tau^{-1}\gamma u + \tau^{-1}\mathrm{S}\big[Ku + X\big] \tag{1}$$

Note that this expression potentially allows for space-dependent model parameters. The steady-state(s) u^* can be obtained by setting the time-derivative and noise amplitude σ to zero and solving the resulting steady state (matrix) equation:

$$\mathrm{S}\big[Ku^* + X\big] = \gamma u^* \tag{2}$$

This equation does not have an analytic, exact, closed form solution (in fact, it doesn't even necessarily have a solution. The sigmoid function is bound between -1 and 1, but γu^* is not). Furthermore, in the context of a whole-brain model on a mesoscopic connectome, the equation is very high dimensional (twice the number of vertices n, with $n \sim 18000$ in our case). This makes a brute-force numerical approach to the determination of steady states computationally inefficient, especially because it would have to be repeated for each parameter set under examination.

If we restrict our analysis to *spatially homogeneous* steady states and *space-independent* model parameters, the steady state equation simplifies to the following 2-dimensional system, rather than the original $2n$-dimensional Eq (2):

$$\frac{1}{1 + exp(\alpha_{IE}I^* - \alpha_{EE}E^* - P)} = d_E E^* \tag{3}$$

$$\frac{1}{1 + exp(\alpha_{II}I^* - \alpha_{EI}E^* - Q)} = d_I I^* \tag{4}$$

Solutions to this 2-dimensional system can be rapidly obtained numerically for any set of parameters. The biologically valid steady state(s) of the model are given by the solutions (E^*, I^*) with $E^*, I^* \in [0, 1]$, since E and I here represent the fraction of active neurons within the respective population. Once a valid steady state is obtained, its stability can be determined through the Jacobian eigenspectrum, and verified with numerical simulations.

To obtain the Jacobian eigenspectrum and determine the stability of a steady state, we linearize the model equations for a small perturbation $(u - u^*)$ about the steady state. For the Wilson-Cowan model (Eq (1)),

we have:

$$\frac{du}{dt} = -\tau^{-1}\gamma u + \tau^{-1}\mathrm{S}\big[Ku + X\big] = F(u) \approx F(u^*) + \frac{\partial F(u^*)}{\partial u}(u - u^*) \tag{5}$$

With $F(u^*) = 0$ by definition since u^* is a steady state. The Jacobian at the steady state is then:

$$J(u^*) = \frac{\partial F(u^*)}{\partial u} = -\tau^{-1}\gamma + \mathrm{Diag}\left(\tau^{-1}\mathrm{S}'[Ku^* + X]\right) K \tag{6}$$

To simplify this expression and allow further analytic progress, we apply the property of the sigmoid derivative $\mathrm{S}'[x] = \mathrm{S}[x][1 - \mathrm{S}[x]]$; the steady state equation $\mathrm{S}\big[Ku^* + X\big] = \gamma u^*$; and finally denoting with $\circ$ the Hadamard (element-wise) product we obtain:

$$J(u^*) = -\tau^{-1}\gamma + \mathrm{Diag}\left(\tau^{-1}\gamma u^* \circ (1 - \gamma u^*)\right) K \tag{7}$$

We have thus obtained a general expression for the Jacobian of the Wilson-Cowan model on graphs, which holds also for non-homogeneous steady states and/or space-dependent parameters. In order to evaluate the linear stability of any steady state, it is sufficient plug in the steady state solution to Eq (2) in Eq (7), and study the eigenspectrum of the resulting Jacobian.

On a mesoscopic human connectome graph such as the one we use here, the general Jacobian of Eq (7) is a dense matrix with more than 10^8 elements. Its eigenspectrum can be calculated numerically, but such a computation is not particularly fast, and has to be repeated for each steady state of each parameter set under examination. However, we restrict the problem to *homogeneous* steady states and *space-independent* model parameters, it is possible to use the properties of the graph Laplacian to obtain an analytic expression for the Jacobian eigenspectrum, which can be computed extremely quickly[1]. These assumptions can be relaxed to allow for eigenmode-dependent[2] model parameters. Define the scalar, steady-state-dependent parameters:

$$a = d_E E^*(1 - d_E E^*), \qquad b = d_I I^*(1 - d_I I^*). \tag{8}$$

We can use the general Jacobian of Eq (7), with Gaussian kernels, to write explicitly the *linearized* Wilson-Cowan equations for the time-evolution of a perturbation about a homogeneous steady state:

$$\tau_E \frac{dE}{dt} = -d_E E + a\alpha_{EE} U e^{\sigma_{EE}^2 \Lambda/2} U^T E - a\alpha_{IE} U e^{\sigma_{IE}^2 \Lambda/2} U^T I + \sigma \xi_E \tag{9}$$

$$\tau_I \frac{dI}{dt} = -d_I I + b\alpha_{EI} U e^{\sigma_{EI}^2 \Lambda/2} U^T E - b\alpha_{II} U e^{\sigma_{II}^2 \Lambda/2} U^T I + \sigma \xi_I \tag{10}$$

Applying the graph Fourier transform U^T, the equations are diagonalized. Each eigenmode of the graph Laplacian therefore behaves independently as a 2-dimensional linear system, with the Jacobian for the kth eigenmode being:

$$J_k = \begin{bmatrix} -\frac{d_E}{\tau_E} + \frac{a}{\tau_E}\alpha_{EE} e^{\sigma_{EE}^2 \lambda_k/2} & -\frac{a}{\tau_E}\alpha_{IE} e^{\sigma_{IE}^2 \lambda_k/2} \\ \frac{b}{\tau_I}\alpha_{EI} e^{\sigma_{EI}^2 \lambda_k/2} & -\frac{b}{\tau_I}\alpha_{II} e^{\sigma_{II}^2 \lambda/2} - \frac{d_I}{\tau_I} \end{bmatrix} \tag{11}$$

[1] These assumptions are required because generally space-dependent parameters would be expressed by a non-constant diagonal matrix, that would not commute with the graph Fourier transform U^T.

[2] Eigenmode-dependent parameters would be expressed by a diagonal matrix in the graph Fourier domain, therefore by definition commuting with U^T.

The Jacobian eigenvalues can then be computed directly as:

$$\lambda_{1,2}^{J_k} = \frac{\mathrm{Tr}\left(J_k\right) \pm \sqrt{\mathrm{Tr}^2\left(J_k\right) - 4\mathrm{Det}\left(J_k\right)}}{2}. \tag{12}$$

From the Jacobian eigenspectrum thus obtained, we can directly infer the stability character of a steady state. Note that in order to obtain meaningful predictions for spatiotemporal observables, the steady state under examination has to be stable, that is, J_k must have no eigenvalues with positive real parts for all λ_k. We have used Gaussian kernels in the derivation, but the result can be straightforwardly generalized to all other kernels. The decoupling of the linearized Wilson-Cowan equations in the graph-Fourier domain has another important consequence: since the dimensionality of the system reduces from n^2 to n (where n is the number of vertices in the graph), very efficient numerical simulations of the linearized equations can be carried out directly in the graph Fourier domain.
