# Supplementary material for: Graph neural fields: A framework for spatiotemporal dynamical models on the human connectome
Source: PLoS Comput Biol. 2021 Jan 28;17(1):e1008310. doi: 10.1371/journal.pcbi.1008310 (PMC7872285; doi:10.1371/journal.pcbi.1008310)
Supplement: Attachment — Submitted filename: Cataldo_rev_PlosCB.pdf [file pcbi.1008310.s016.pdf]

Dear Editor,

this is a well written and elegant paper of theoretical and computational neuroscience, in which the authors have applied methods derived from graph signal processing to neuroimaging data, extracting from them the connectome graph.

In particular, the authors derive and use, for the first time, a weighted graph Laplacian operator in function of graph differential operators, in place of a combinatorial graph Laplacian, used by them in a previous published paper.

This new approach, more adequate when the properties of the cerebral cortex are to be taken into account, is tested on some well known models of the cortex, such as the Wilson-Cowan model. One of the claim of this paper is that, with respect to the continuous spatio-temporal neural field models, this appoach is more efficient in terms of computational cost. The new techniques are clearly developed and formally compared to the neural field approach, along the paper.

The paper could be published as it is, after having inserted the few changes suggested below. Nevertheless, it would become a stronger paper if the authors would make a quantitative (numerical) comparison, in terms of effective computational cost and accuracy, between the graph neural field approach presented and the spatio-temporal continuous neural field approach on some given experimental connectome.

Minor points:

1) Table1 and Table2 and their captions should be made more clear;

2) p 137: ‘we have to linearize eq 13’ should become ‘we have to linearize eq 14’.

3) Results section: this section keeps quoting definitions and results that are in the Methods section. This negatively affects the readability of the article, continuously breaking the flow of the discussion. Considering that in the Methods section there are no quotes related to the Results section, it seems a better natural choice to swap the two sections. If this cannot be done, I would suggest putting in the appendix the results from the Methods section that are quoted in the Results section.

Some typos and suggested improvements:

- p 122, Linear stability analysis paragraph:

"to obtain solutions the steady-state equations" should become "obtain solutions TO the steady state equations"

- p 210, Application to resting-state functional MRI section:

"use it reproduce" should become "use it TO reproduce"

- p 595, Discussion:
"they can are defined" should become "they are defined"

- p 257, Convolution of graph signals: While properties of the spatiotemporal Fourier transform are used, the graph Fourier transform is used. The readability of this section would greatly improve by dedicating a couple of sentences to the reason why the generalized version of the Fourier transform still has the same properties (such as convolution theorem).

- p 456, Graph neural fields; It should be specified that equation (62) is only valid for neural fields of type 2, as it was done for equation (61)
