# Supplementary material for: Graph neural fields: A framework for spatiotemporal dynamical models on the human connectome
Source: PLoS Comput Biol. 2021 Jan 28;17(1):e1008310. doi: 10.1371/journal.pcbi.1008310 (PMC7872285; doi:10.1371/journal.pcbi.1008310)
Supplement: Attachment — Submitted filename: reponse_GNFs_edited.pdf [file pcbi.1008310.s017.pdf]

Dear Editor and Reviewers,

First, we would like to thank you for the positive comments about our work, and for the very constructive reviewing process, which we believe helped us significantly improve the manuscript. We have undertaken a major revision of the manuscript, consisting of the following components:

1) Robustness of application: in a major new addition to Results, we predict the connectome-wide fMRI functional connectivity at mesoscopic vertex-wise resolution (we also added an extra set of 1D analyses and simulations to illustrate the influence of model parameters on model observables).

2) Improved readability: we moved several subsections to SI, shortening the manuscript main text by several pages. We worked on improving consistency, clarity, and reducing redundancy of mathematical notation.

3) Better contextualization: we added references and expanded the text wherever the reviewers requested this.

We would like to remark that, unfortunately, in the automatically-generated submission manuscript, figures are not shown at the original resolution. We kindly ask the reviewers to click the links present in each figure page in order to download the figures at full scale. In supplementary figures S6-S9, we also provide PDFs for the functional connectivity figures at higher resolution than what is possible in the main text because of format/size restrictions. Note also that in the original submission, we included appendices in the main text, which we have now been informed is not the correct practice. Appendices are now provided separately as requested by the Editor.

In the rest of this letter, we provide specific answers to the reviews. We hope that the manuscript is now fully suitable for publication in PLOS Computational Biology. Looking forward to hearing from you.

Best,

Marco Aqil (corresponding author)

Reviewer #1

The paper presents an integration of concepts from graph signal processing and neural field models. In particular, the latter is extended to the former setting. To this reader, the work is original, innovative and has the potential to be leveraged to shed light in to the dynamics of resting state fMRI data through using local and distant backbone connections of the brain. The methodology, although description-wise not so nicely laid

out, is rigorous and provides notable insights for the community. Despite its strengths, the paper has left this reader with two main concerns:

1) The paper is titled heavily in relation to the human connectome, whereas the contents are essentially theoretical derivation of general nature, which are applicable to any type of graph. Although the incorporation of the neural field models into the graph setting does put the work in context in regard to the neural setting, the majority of presented results are on a simplistic 1D graph of a non-neuronal nature. Results of the human connectome are limited to that of a single subject, the most substantial of which is to show that the power spectral density of a single time frame of the subject's resting-state fMRI data can be fitted with the proposed model. To this reader, this extent of results is extensively limited to prove the applicability and robustness of the proposed methodology on fMRI data, and as such, the work lacks substantial evidence for the conclusions made. A number of suggestions are presented in the comments that follow, which the authors may want to consider.

To prove the robustness of the framework, we extended the fMRI results to predict vertex-wise connectome-wide functional connectivity (**Results>Application to resting-state fMRI>Graph neural fields on the human connectome predict the vertex-wise functional connectivity of resting-state fMRI**). However, we also note that this comment appears to suggest that the harmonic spectrum is estimated by "a single time frame of the subject's resting state fMRI"; this is not the case. The computation of the harmonic spectrum does not use a single time frame, but rather the entire resting-state BOLD fMRI time-course.

2) The presentation of the paper, in particular in relation to the mathematical formulations are not consistent and the associated descriptions are generally not concise. A series of errors and issues are listed in the comments that follow. To increase the readership of the work, the authors are highly recommended to consider thoroughly revising the manuscript for brevity.

We thank the reviewer for pointing out specific notational inconsistencies and possible improvements. We address each comment directly (see below).

a) In the paragraph before equation (33), you state: 'the elements of M can be defined in terms of suitably scaled Euclidean distances, geodesic distances over the cortical manifold, or as the lengths of the white matter fibers connecting the vertices.' It may benefit the reader if you could refer to related works for the listed scenarios, for instance, for the third one: https://doi.org/10.1109/TMI.2013.2271486, which defines intra-cortical graph edges based on the surface mesh in a similar way as done in your work. The work is also probably the first to present the idea designing hybrid graph with local and distant edges, and also uses non-binary edges, see equation (6).

Thanks for pointing out this reference, which we have added to the text.

b) As mentioned earlier above, results on a single subject is just too little. Can you extend results shown in Figure 7 across multiple subjects, potentially on the HCP100 data? If you normalize the fMRI graph signals to unit norm, and use the normalized graph Laplacian, which has its eigenvalues bounded within [0,2], you may then overlay the resulting power spectra, or the cumulative sum for instance as in Fig. 3(a) in: https://doi.org/10.1109/ISBI45749.2020.9098667, across subjects. Moreover, as your model is not specific to resting-state data, it can be intuitive to see the same results also on the HCP task data, at least for a subset of the subjects if computationally burdensome.

Unfortunately the co-author that carried out the preprocessing of the anatomical data was not available to do this for additional subjects. While we understand the reviewer's concern towards the single-subject application, we note that the harmonic power spectrum only shows minor variations across subjects, while the overall shape is preserved (Atasoy et al. 2017), hence we can reasonably expect that the model should be able to fit the harmonic power spectrum of any subject, with minor or no changes in parameters. We believe that the possibility of studying individual subject-specific models rather than group-data is a strength of our framework. In-depth analysis of the subtle changes in harmonic spectrum across subjects, their biological meaning, and how these are reflected in model parameters could definitely be the subject of future work, but perhaps beyond this initial presentation. The application to task data is definitely another interesting direction of future application. To prove the robustness of the framework, and the significance of the harmonic power spectrum, we now show that the model obtained by fitting the harmonic power spectrum is also able to predict single-subject fMRI functional connectivity with a high level of detail (**Results>Application to resting-state fMRI>Graph neural fields on the human connectome predict the vertex-wise functional connectivity of resting-state fMRI**).

c) The FC matrices shown in Figure 5 are shown at a resolution that cannot be readily compared with each otehr. Could you please provide a zoomed inset of a region around the diagonal on both matrices, to enable pixel-by-pixel comparison of the two at the zoomed inset? Moreover, to better convey that the two matrices are very similar, you can show the difference between the two, and again, show a zoomed in region around the diagonal. Lastly, the colormap used does not nicely reflect positive-negative values with a nice separation of 0; in particular, you state: line 202: 'The non-local edge also creates a visible increase in the functional connectivity between the nodes involved, and a change in the pattern in neighboring nodes' which is not at all as visible as you state. If you modify your colormap, for instance to show 0 as white, things should become much more visible. In doing so, you may want to use the following in matlab: RdBu = flipud(cbrewer('div','RdBu',101)); colormap(RdBu); for which you need to download and addpath this package:
https://www.mathworks.com/matlabcentral/fileexchange/34087-cbrewer-colorbrewer-schemes-for-matlab

Following the reviewer's request, we have changed the colormap and colorscale of the FC matrices to make differences more apparent. We also note that unfortunately the resolution of figures shown in the PLOS-formatted submission PDF does not correspond to the full resolution of submitted figures. Full-resolution figures can be obtained by clicking the download link on each figure page in the submission PDF. We kindly ask the reviewers to disregard the low-quality preview figures shown in the submission PDF, and to please download the full-resolution figures by clicking on the link in each figure page. In S1File we provide an even higher resolution version of the new functional connectivity figures.

d) On line 526 you state: 'intra-cortical edges are weighed by they 3D Euclidean distance'. Can you more specifically state how you define these weights? In particular, do you divide or multiply by the Euclidean distance? (Also note the typo: they > their.) But aside from this, I found your approach in scaling of the non-local edge weights very intuitive and an interesting way to account for intra-cortical propagation.

Following the reviewer request, we now clarified in the text how the weighing takes place (**Methods>Data preprocessing and connectome graph construction**):

the graph. The main difference with previous studies analyzing brain activity in terms of the anatomical connectome graph Laplacian [11] is that instead of constructing the combinatorial (binary) graph Laplacian, here we construct a distance-weighted graph Laplacian (Eq (19-22)). This allows us to take into account physical distance properties of the cortex that are relevant for graph neural fields, and that are otherwise lost. Specifically, for an local surface edge between vertices i and j, the element M_{ij} of the distance matrix M is defined as their 3D Euclidean distance; for a non-local white-matter edge, M_{ij} is defined as the distance along the respective DTI fiber path, divided by a factor of 200. This value is chosen to reflect the myelination of white matter fibers, which is known to allow neural activity to propagate at speeds ~ 200 times greater in white matter fibers, in comparison with local surface propagation [25]. Resting-state BOLD

e) On line 607 you state: 'Graph neural fields can naturally take into account important physical properties such as cortical folding, hemispheric asymmetries, non-homogeneous structural connectivity, and white matter projections, with a minimal amount of computational power.' This is indeed interesting, but can you please elaborate a bit on how this is achievable?

Following the reviewer request, we elaborate on this point in the **Discussion**:

Graph neural fields have the advantage of being implementable directly on multimodal structural connectomes obtained from MRI and DTI, thereby minimizing anatomical approximations, and being limited in this sense only by the quality and resolution of the available structural data. The cortex in graph neural field need not be a flat or spherical manifold, but can reflect the specific anatomy of each subject, allowing in-depth analyses of the effects of individual anatomical differences on functional activity; such analyses can then be compared across subjects thanks to the common language provided by the connectome harmonics. Graph neural fields can take into account important physical properties of the cortex, such as folding, non-uniform thickness, hemispheric asymmetries, non-homogeneous structural connectivity, and white-matter projections, since all these anatomical features can be absorbed in the distance-weighted graph Laplacian. In

[…]

Graph neural fields come equipped with computationally efficient analytic and numerical tools. The CHAOSS method allows fast computation of quantities such as the harmonic-temporal spectra or connectivity matrices without resorting to numerical simulations, which are enormously more computationally expensive than the direct evaluation of analytic expressions. This implies that optimization of model parameters (for example to fit an observable quantity such as the harmonic spectrum, as we do here) can be carried out without the computational burden of numerical simulations. Furthermore, linear or linearized graph neural field equations are diagonalized by the graph Fourier transform, allowing very efficient numerical simulations in the graph Fourier domain. For a graph neural field on a connectome with n vertices, carrying out numerical simulations in the graph Fourier domain reduces the dimensionality by a factor of n, which is a vast improvement for high-resolution connectomes. Hence, graph neural field analysis (CHAOSS) and numerical simulations (linear or linearized models in the graph Fourier domain) can be carried out with a minimal amount of computational power.

f) The connectome model, when used for the sake of representation as the backbone on which fMRI data are realized, should be interpreted with care, in particular, in light of, firstly, limitations with tractography in regards to false positive and true negatives, and secondly, lack of projection fibers in the model that connect the cortex and subcortical structures such as the thalamus, basal ganglia, and spinal cord and the cerebellum. The concern is not only lack of connections, but in fact lack of vertices representing these structures. I understand that the presented work is not expected to address these concerns, but I believe these concerns deserve be mentioned, at least briefly, in the Discussion, to inform the reader.

We agree with the reviewer that this concern is worth mentioning to inform the reader. We explicitly pointed this out among the other limitations in the **Discussion:**

> graph edges within specific ROIs or specific subsets of vertices. Second, it is important to point out that our approach is subject to the limitations of tractography in regards to false positive and true negatives; and that the connectome used here does not include subcortical structures, nor projections between the cortex and subcortical structures.

g) The notation P(k) used in the y-axis label of Figure 7 seems not to appear in text, but you have $H_{p}(k)$, though I do not perfectly follow what would be the 'neuronal population' in this example figure if relevant.

We thank the reviewer for pointing out this imprecision, which has now been corrected. The neuronal population would be the excitatory population of the Wilson-Cowan model ($H_E(k)$, Eq 14).

h) On line 594 you state that: 'Whole-brain models that incorporate short-range connectivity are referred to as surface- based because they can are defined either on high-resolution surface-based representations of the cortex'. However, models that incorporate short-range connectivity can also be volumetric, e.g. see: [whole brain graph] https://doi.org/10.1016/j.neuroimage.2020.116718, [white matter graph] https://doi.org/10.1109/ISBI45749.2020.9098582, [gray matter graph] https://doi.org/10.1016/j.neuroimage.2015.06.010. There is no need to refer to these works as their aim differs in nature from that in your work, aside from the first one to some extent. The concern is with the sentence that is not quite accurate.

We adjusted the text following the reviewer's indication.

i) At the C Elegans statement, line 668, you may find the following work of relevance to refer to: https://doi.org/10.1162/netn_a_00084

Reference added.

j) To give the reader a more recent comprehensive review of GSP and its applications consider citing the following: https://doi.org/10.1016/j.dsp.2020.102802

Reference added.

k) You may want to state that the filtering operation of graphs using any desired spectral kernel can be efficiently implemented using polynomial approximation scheme which obviates the need to diagonalize the Laplacian matrices, which in your setting of 18K nodes, although feasible, but still quite computationally cumbersome to get all the eigenmodes. If you decided to mention such an option as outlook, you may find this recent preprint of interest to refer to as it provides a nice overview of available techniques: https://arxiv.org/abs/2006.11220

Thanks for this suggestion. We have added the reference.

l) In the paragraph above equation (75), it states: ‘Because of the independence of eigenmodes,...’. I suppose you mean 'orthogonality' of the eigenmodes? (Also note that, i) the type at the end of the paragraph: spectrum. > spectrum: --- ii) sometimes you use: when presenting equations and sometimes not; please consider making the presentation consistent)

Following the reviewer’s suggestion, we clarify this point in the text. In short, because the eigenmodes are orthogonal, the graph Fourier transform can be used to diagonalize linear or linearised model equations with space-independent model parameters. Consequently, in such cases, the dynamic of each graph Laplacian eigenmode can be described independently, as a p-dimensional linear system, where p is the number of neuronal populations types in the model (p=2 for the Wilson-Cowan model).
We adjusted the notation for presenting equations throughout the text for consistency.

m) For constants, you sometimes use lower case letters, like n that specifies the number of eigenmodes, and sometimes use uppercase, like N that specifies the number of neuronal populations.

Following the reviewer’s suggestion, we changed the symbol for the number of neuronal populations to lowercase ‘p’, and tried to specify in the text as clearly and as unambiguously as possible which variables are scalars, vectors, or matrices.

n) You redefine Dt multiple times and in an inconsistent way. Firstly, this is a very inconcise, and disturbing for the reader. Secondly, you sometimes refer to it as: 'where Dt is A temporal differential operator', and sometimes as: 'where Dt is THE temporal differential operator'. Moreover, you apparently use three different notations for temporal differentiation, which is quite sloppy: i) \dot{.}, ii) \frac{\partial .}{\partial t}, iii) Dt .

Following the reviewer suggestion, we removed the \dot{} notation for temporal derivatives entirely since it is equivalent to d/dt and hence redundant. Concerning D_t, we think the confusion arises from the fact that other authors have used D_t to signify the first-order temporal derivative, whereas in our text D_t is a general placeholder for any linear temporal differential operator (e.g. in the general graph equation D_t u(t) = \Delta u(t); setting D_t = d/dt gives diffusion, D_t=d^2/dt^2 gives the wave equation, D_t = d/dt +d^2/dt^2 gives the damped-wave equation). We updated the text to clarify that the notation D_t represents a general differential operator.

o) In describing graphs, sometimes you use the term ‘nodes’ and sometimes ‘vertices’. Please stick with one.

Changed all mentions of ‘node’ or ‘nodes’ to ‘vertex’ or ‘vertices’ throughout the text.

p) Table 1 should either be placed earlier, or be referred to early on in the section to prevent losing the reader.

Thanks for this suggestion. Table 1 is now placed earlier in the section.

q) It would be much better to present the diagonalization of the Laplacian and related notations (lines 166-117) before/after eq. (2); then you can skip the related descriptions of the degree matrix, after eq (4), and U matrix in eqs. (11)-(12). You are also repeating the diagonalization relation before eq. (38). Please do thoroughly re-read your paper, throughout, as there are many such instances of unnecessary and redundant repetitions.

Following the suggestion, we presented the diagonalization of the Laplacian early in the text and removed redundant repetitions.

r) You use multiple notations for vectors: bold lower case, upper case, bold upper case, for instance ‘d’, ‘K’ and ‘X’ in eq. (13). This also happens in the other equations. This is sloppy and super confusing for the reader.

Following the reviewer’s suggestion, we removed all bold-vector symbols to make things more consistent and clearer for the reader (note also that the full linear stability analysis is now in SI).

s) The sentence after eq. (17) is either incomplete or badly written if it is linked to (17).

Following the reviewer’s suggestion, we have improved the phrasing of sentences near eq. 17 (now in SI).

t) In the description after eq. (18), you denote S as S(.) whereas in the definition you use S[.].

Corrected in the text.

u) Sometimes you say 'eigenmode k' and sometimes 'kth eigenmode'. Please consider making the description consistent and stick to one scheme.

Corrected in the text.

v) Some other typos:
- After equation (73): Where > where
- After equation (74): where 1/2\pi is. > this is obvious, so you may drop it.
- There are many repetitive sentences. For instance: ‘where n/N is…’. It should be sufficient to define these once.
- Line 106: ‘…5s of…’ > ‘…5 seconds of…’
- Line 515: (i; j)^{th} > is th needed?
- Line 579: varies > various

- Comma missing at the end of equations (10), (24), (67), ?
- Full stop missing at the end of equation (23), (17), (22), (20), (18), (19), (40), ?
- After eqs. (4), (10), etc. start sentence with 'where'; e.g. 'where' \Delta is..
- Before eq (11): are matrices of size (n; n) > are $n \times n$ matrices.
- Page 8, second line: solutions the steady... > solutions TO the steady...
- Page 9, first sentence: abut > about

Corrected in the text.

Reviewer #2

This paper introduces a graph neural field approach to describe whole brain neural activity which allows to derive spectra and also functional connecitivity. The authors use concepts from neural fields and graph signal processing to achieve this. Overall, I am very enthusiastic about this work and it definitely contributes to the growing interest and role of the eigenmodes to brain activity and connectivity. The math is rigorous and can be clearly followed. I do have a few concerns.

Major concerns

My suggestion would be to reorder and shorten the paper. The paper in its current form is quite long. The current ordering of the sections is also confusing and not according to the journal's guidelines. I would suggest to have the following order: introduction, results, discussion, methods. I think there are parts of the paper that were necessary as initial sanity checks, such as "damped wave equation on the human connectome" in the result section and "Reaction-diffusion neural activity models". Though relevant for following the reasoning and steps in the paper, these sections also quite distract from the main storyline of the paper. I understand the role of the linear stability analysis applied to the Wilson-Cowan equations. However, this type of analysis on Wilson-Cowan equations on a network is not new (see (Tewarie et al., 2019)). I would suggest to put all these/or part of these sections to the supplemental material.

Thanks for these suggestions. We reordered the main sections of the manuscript as: introduction, results, discussion, methods. Apologies for the previous ordering which indeed did not follow the journal guidelines. Following the reviewer's suggestion, in order to shorten the paper and avoid distracting the reader, we moved "damped wave equation on the human connectome", "Reaction-diffusion neural activity models", and the "Wilson-Cowan linear stability analysis on graphs", to the supplementary information.

The authors use a Gaussian Kernel for their Wilson-Cowan model based analysis. The authors do illustrate the possibilities of the kernels (see method section). Maybe I haven't noticed it, but I haven't seen any analysis on the influence of these different kernels. The choice of the kernel can actually have dramatic influence on the stability of the steady states and the model's bifurcation structure (Atay and Hutt, 2004; Hutt and Atay, 2005; Shamsara et al., 2019). The authors have merely used the Gaussian Kernel with one value for the standard deviation. It is not clear to me how this standard deviation was the result of some optimization. Nor is it clear to me whether this Kernel also optimized the resemblance of functional connectivity with empirical data. Why did the authors not use a range of sigma? It would be interesting if the authors can discuss or speculate on the usage of different kernels for E and I cells, for some I cells, there is evidence of lateral inhibition, which would advocate for example for a Mexican hat kernel for I population (in the Wilson-Cowan example).

Following the reviewer interesting suggestion, we clarified our choice of kernels and added simulations, showing that even just changing the size of one of the four kernels can significantly alter the model dynamics and stability of the steady state. Importantly, we note that we did not use just one value for the standard deviation. Rather, each of the four Gaussian kernels describing population interactions in the Wilson-Cowan model (E-E, E-I, I-E, I-I) has a *different* standard deviation, hence we do use a range of different sigmas. Effectively, this can give rise to lateral inhibitory interactions without the use of Mexican hat kernels, which would indeed be required if the model only comprised a single population (some authors have described such versions of the Wilson-Cowan model). The standard deviations of the four Gaussian kernels (and all other model parameters, S2 Table) for analysis and simulations on the connectome were obtained by fitting the model harmonic power spectrum to the fMRI harmonic power spectrum.

It is not clear to me how well the prediction of the model is for empirical functional connectivity patterns from fMRI. The authors do show how well the modeled spectra match the empirical one in Figure 7. However, equally important is whether modeled activity patterns or functional connectivity patterns match the empirical data. I doubt whether functional connectivity patterns such as in figure 6 match empirical fMRI based connectivity matrices.

Following the reviewer's suggestion, we expand the Results by predicting the functional connectivity for the connectome-wide Wilson-Cowan model. Indeed, the functional connectivity pattern on a connectome-wide graph looks very different from the patterns obtained on a simple one-dimensional graph. We were able to reproduce fMRI functional connectivity with a high level of detail, without fitting the fMRI FC, but only the harmonic power spectrum (**Results>Application to resting-state fMRI>Graph neural fields on the human connectome predict the vertex-wise functional connectivity of resting-state fMRI**).

I cannot find any mentioning of distance dependent delays. Can the authors discuss or mention if distance dependence delays for the propagation along white matter tracts can be implemented in their approach ((Alswaihli et al., 2018)), and otherwise comment how their distance weighting would make this less important? Could the authors please clarify how the distance was set for the Wilson-cowan based simulations on page 11. Did the authors use the distance along the cortical surface for local edges? Distance along the white matter tract for long range connections? If not, I would suggest the authors to implement this. I do not understand on page 11 that the non-local connections are merely based on DTI data? Cortico-cortical connections do not necessarily go through white matter tracts, but can propagate through connections between infra/supragranular layers in the cortex.

Following the reviewer's suggestion, we added a paragraph to the discussion of the revised manuscript in which we discuss how delays can be incorporated into graph neural fields.

As suggested by the reviewer, we indeed used distance along cortical surface and path-distance along white matter tracts for local and non-local edges respectively. This is elaborated on in the Methods subsection "Data preprocessing and connectome graph construction".
The resolution of our connectome does not allow distinguishing multiple cortical layers, hence in this context we have used 'cortico-cortical' as a synonym of white matter connections, and 'intra-cortical' as a synonym of local connections on the surface. We have now corrected this in the text to avoid confusion. Specific cortico-cortical connections between infra/supragranular layers could be taken into account in future studies employing multi-layer connectomes. That is a very interesting possible direction of future development.

Minor concerns

I would put "eigenmodes" in the keywords, or even in the title.

Following the reviewer suggestions, we will put eigenmodes in the keywords.

Are sigma in equations (9) and (10) the same as sigma in (11) and (12), i.e. to they correspond to the standard deviation of Gaussian Kernels.

Yes, they are, indeed the four different sigma parameters \sigma_EE, \sigma_IE, \sigma_EI, \sigma_II determine the size of Gaussian kernels describing population interactions.

Maybe I overlooked this, but please explicitly mention what the definition of d_j is in (33).

d_i in Eq 33 (original manuscript, now the numbering of equations has changed because of major revision) is the first-order directional derivative. (Now Eq 19).

The authors could consider to rename some state variables. The matrix U contains the eigenvectors of the graph Laplacian. At the same time there is state variable u(t). When people scan your paper, they can confuse u to be columns of U. I had this confusion too when I scanned the paper at first. I know that u(x,t) is often used in the field of neural fields to describe the state variables, maybe you could consider to rename the eigenvectors of the graph Laplacian?

This is an unfortunate coincidence: the notation U^T\LambdaU has also been used in several previous studies to describe the eigendecomposition of the graph Laplacian. And as the reviewer points out, u(t) is often used as the state variable in graph neural fields. We attempt to make sure this ambiguity is resolved in the text by clearly pointing out the meaning of U and u(t) early in the text (eq 6,10).

I assume equations (62), (65), (67), (69) refer to type 2 neural field equations. Please explicitly mention to make it easy for the reader.

The reviewer is correct, and we now make this explicit for clarity.

I think this statement in the discussion needs references: “This prevents studying the mechanisms underlying a large class of cortical activity patterns that have been observed in experiments, including traveling and spiral waves, sink-source-dynamics as well as their role in shaping macroscopic dynamics.”

We added a reference to Breakspear’s review of neural masses and neural fields. The statement is a direct consequence of the fact that in ROI-based models, the spatial extent of each ROI is disregarded, and each is treated as a point despite having a size of the order of several cm^3. Hence mesoscopic activity propagation cannot be studied.

There is a spelling error in the first sentence of the 4th paragraph in the discussion:” based because they can are defined either on high-resolution”. This is either “are” or “can”.

Corrected spelling.

I would suggest to use consistent terminology, it is either nodes and links (networks) or vertices and edges (graphs). The authors use edges and links both at the same time with vertices.

Corrected terminology: all mentions should now refer to vertices and edges.

Some recent literature deserves to be mentioned in the context of eigenmodes expression in functional MRI or EEG data, see recent papers: (Glomb et al., 2020; Preti and Van De Ville, 2019; Tewarie et al., 2020). How much different is the damped wave equation on the connectome compared to (Caputo et al., 2017).

em to the text. The wave equation studied by Caputo et al. is a nonlinear variant of the wave equation: d^2/dt^2 u = \Delta u - u^3, whereas the damped-wave equation we study is linear but contains an additional diffusion term, and reads a d^2/dt^2 u + d/dt u = \Lambda u. Furthermore, as far as we understand, they do not make use of a distance-weighted graph in their study.

Alswaihli, J., Potthast, R., Bojak, I., Saddy, D., and Hutt, A. (2018). Kernel reconstruction for delayed neural field equations. J. Math. Neurosci. 8, 3.

Atay, F. M., and Hutt, A. (2004). Stability and bifurcations in neural fields with finite propagation speed and general connectivity. SIAM J. Appl. Math. 65, 644–666.

Caputo, J.-G., Khames, I., Knippel, A., and Panayotaros, P. (2017). Periodic orbits in nonlinear wave equations on networks. J. Phys. A Math. Theor. 50, 375101.

Glomb, K., Queralt, J. R., Pascucci, D., Defferrard, M., Tourbier, S., Carboni, M., et al. (2020). Connectome spectral analysis to track EEG task dynamics on a subsecond scale. Neuroimage 221, 117137.

Hutt, A., and Atay, F. M. (2005). Analysis of nonlocal neural fields for both general and gamma-distributed connectivities. Phys. D Nonlinear Phenom. 203, 30–54.

Preti, M. G., and Van De Ville, D. (2019). Decoupling of brain function from structure reveals regional behavioral specialization in humans. Nat. Commun. 10, 1–7.

Shamsara, E., Yamakou, M. E., Atay, F. M., and Jost, J. (2019). Dynamics of neural fields with exponential temporal kernel. arXiv Prepr. arXiv1908.06324.

Tewarie, P., Abeysuriya, R., Byrne, Á., O’Neill, G. C., Sotiropoulos, S. N., Brookes, M. J., et al. (2019). How do spatially distinct frequency specific MEG networks emerge from one underlying structural connectome? The role of the structural eigenmodes. Neuroimage 186, 211–220.

Tewarie, P., Prasse, B., Meier, J. M., Santos, F. A. N., Douw, L., Schoonheim, M., et al. (2020). Mapping

functional brain networks from the structural connectome: relating the series expansion and eigenmode approaches. Neuroimage 216, 116805. doi:https://doi.org/10.1016/j.neuroimage.2020.116805.

Thanks for the references. We have added them to the text.

Reviewer #3

Dear Editor, this is a well written and elegant paper of theoretical and computational neuroscience, in which the authors have applied methods derived from graph signal processing to neuroimaging data, extracting from them the connectome graph.

In particular, the authors derive and use, for the first time, a weighted graph Laplacian operator in function of graph differential operators, in place of a combinatorial graph Laplacian, used by them in a previous published paper.

This new approach, more adequate when the properties of the cerebral cortex are to be taken into account, is tested on some well known models of the cortex, such as the Wilson-Cowan model. One of the claim of this paper is that, with respect to the continuous spatio-temporal neural field models, this appoach is more efficient in terms of computational cost. The new techniques are clearly developed and formally compared to the neural field approach, along the paper.

The paper could be published as it is, after having inserted the few changes suggested below. Nevertheless, it would become a stronger paper if the authors would make a quantitative (numerical) comparison, in terms of effective computational cost and accuracy, between the graph neural field approach presented and the spatio-temporal continuous neural field approach on some given experimental connectome.

Although we find this an interesting suggestion, we feel it is outside the scope of this study and prefer to emphasize the conceptual contribution of our study.

Minor points:

1) Table1 and Table2 and their captions should be made more clear;

Following the reviewer’s suggestion, we attempted to clarify the captions of Table 1 and Table 2.

2) p 137: ‘we have to linearize eq 13’ should become ‘we have to linearize eq 14’.

Sentence changed. Note that linear stability analysis is now in SI.

3) Results section: this section keeps quoting definitions and results that are in the Methods section. This negatively affects the readability of the article, continuously breaking the flow of the discussion. Considering that in the Methods section there are no quotes related to the Results section, it seems a better natural choice to swap the two sections. If this cannot be done, I would suggest putting in the appendix the results from the Methods section that are quoted in the Results section.

In the revised manuscript, we have tried to make the Results section significantly more readable.

Some typos and suggested improvements:

- p 122, Linear stability analysis paragraph: "to obtain solutions the steady-state equations" should become "obtain solutions TO the steady state equations"
- p 210, Application to resting-state functional MRI section: "use it reproduce" should become "use it TO reproduce"
- p 595, Discussion: "they can are defined" should become "they are defined"
- p 257, Convolution of graph signals: While properties of the spatiotemporal Fourier transform are used, the graph Fourier transform is used. The readability of this section would greatly improve by dedicating a couple of sentences to the reason why the generalized version of the Fourier transform still has the same properties (such as convolution theorem).
- p 456, Graph neural fields; It should be specified that equation (62) is only valid for neural fields of type 2, as it was done for equation (61)

We addressed these points in the text.
