# Supplementary material for: Correction: Graph neural fields: A framework for spatiotemporal dynamical models on the human connectome
Source: PLoS Comput Biol. 2022 Jun 1;18(6):e1010224. doi: 10.1371/journal.pcbi.1010224 (PMC9159585; doi:10.1371/journal.pcbi.1010224)
Supplement: S1 Table — This parameter set was obtained by a qualitative comparison of the Wilson-Cowan model’s harmonic and temporal spectra with empirical data, and used to illustrate how graph properties affect neural field dynamics in one dimension. (PDF) [file pcbi.1010224.s001.pdf]

Parameter set for 1D analysis and simulations.

Marco Aqil, Selen Atasoy, Morten L. Kringelbach, Rikkert Hindriks

November 20, 2021

**Parameter**	**Value**	**Units (S.I)**
τ_E	$2.415 \cdot 10^{-1}$	s
τ_I	$5.227 \cdot 10^{-1}$	s
σ_{EE}	$4.577 \cdot 10^{-3}$	m
σ_{IE}	$1.704 \cdot 10^{-3}$	m
σ_{EI}	$9.377 \cdot 10^{-3}$	m
σ_{II}	$2.239 \cdot 10^{-1}$	m
d_E	10^2	-
d_I	9.430	-
α_{EE}	$6.886 \cdot 10^2$	-
α_{IE}	$7.903 \cdot 10^1$	-
α_{EI}	$9.972 \cdot 10^2$	-
α_{II}	$1.223 \cdot 10^2$	-
P	3.824	-
Q	7.120	-
σ	10^{-7}	-
